# The Practitioners' Bookshelf

**Published:** 1893-02-04

**Authors:** 


### THE PRACTITIONERS' BOOKSHELF.

### "THE DISEASES OF CHILDREN: MEDICAL AND SURGICAL."*

This work is one of the most practical and complete treatises on diseases of children that have been published, and we think that these two features, very essential in a work of this kind, are the result of the collaboration of a physician and surgeon. That it has been appreciated by the profession is shown by the fact that a second edition has been required in such a short time. The whole of the work has been thoroughly revised, and several of the sections have been entirely rewritten. The addition of many clinical reports of interesting cases, and forty new woodcuts—most of them original—have been added. We can fully endorse the authors' claim that the work has been kept abreast of the times.

The opening chapters on the physiology of infancy and childhood and the hygiene and diet of infants and children show this to be the work of a physician who has a very practical experience of these subjects, as well as the theoretical and so-called "scientific" aspects of these important departments, but it is hardly necessary to enter into details of a work which has become so well known to the profession. It is written throughout in a plain, clear, and pleasant style.

The surgical portion of this work, too, deserves the highest praise. Mr. Wright, at the Pendlebury Children's Hospital (Manchester), with 140 beds, has enormous opportunities of studying the surgical diseases of children. It is a practical book of great value; it does not go minutely into pathology, only enough to make clear the clinical features of the disease. It is full of hints on medical and surgical treatment. The first edition was well illustrated, and many more drawings have been added to this. All the latest information, both from foreign and British journals, seems to have been collected and utilized in the production of this work. Talipes is very fully described, and its treatment clearly indicated. The "artificial muscle" plan of treating club foot is described and illustrated. The very important subject of tracheotomy receives careful and full attention, and we are instructed, so far as we can be in a book, how to overcome its difficulties. There are two good illustrations of club hand, and an interesting attempt to treat this condition (which is generally due to deficient development of one of the bones of the forearm) by transplanting some bone from another child into an incision between the muscles of the forearm. "The wound," says Mr. Wright, "healed perfectly, and the bone was growing at the time of the child's death from an independent cause two or three weeks later. The position of the hand was much improved." We know of no book which so well describes the rickety deformities of children—the description being thoroughly illustrated. So good, indeed, are the illustrations throughout, that we frequently take the book to our demonstrations at a children's hospital to show to the students some feature in a case. Both to the surgeon and the physician the book will prove one of very great value, and we very heartily commend it to the notice of all students and practitioners.

* "The Diseases of Children: Medical and Surgical." By Henry Ashby, M.D.Lond., F.R.C.P., and G. A. Wright, B.A., M.B.Oxon., F.R.C.S.Eng. Second edition. Illustrated. (London: Longmans, Green, and Co., 1892.)

